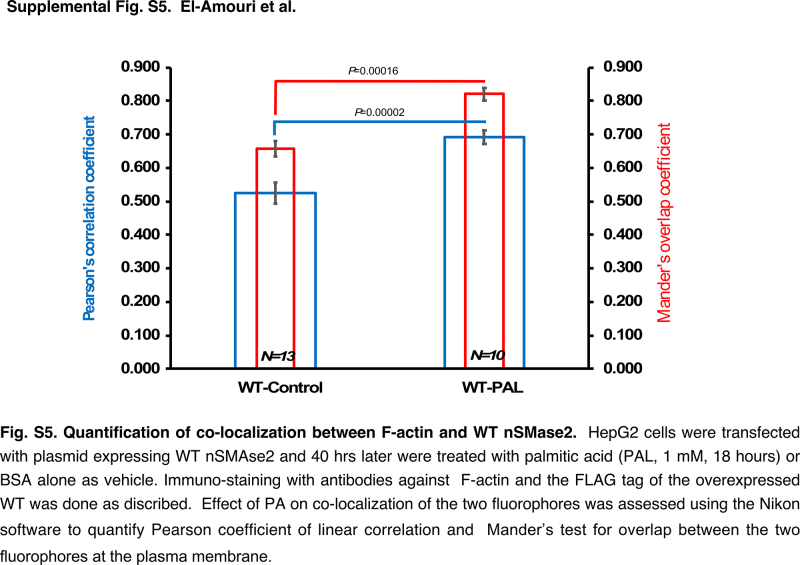# Regulated translocation of neutral sphingomyelinase-2 to the plasma membrane drives insulin resistance in steatotic hepatocytes

**DOI:** 10.1016/j.jlr.2023.100435

**Published:** 2023-08-26

**Authors:** S. El-Amouri, A. Karakashian, E. Bieberich, M. Nikolova-Karakashian

**Affiliations:** Department of Physiology, University of Kentucky College of Medicine, Lexington, KY, USA

**Keywords:** ceramides, sphingolipids, plasma membrane, palmitoylation, insulin resistance, neutral sphingomyelinase 2, smpd3, palmitic acid, Akt, hepatocytes

## Abstract

Obesity-associated diabetes is linked to the accumulation of ceramide in various organs, including the liver. The exact mechanisms by which ceramide contributes to diabetic pathology are unclear, but one proposed scenario is that ceramide accumulation may inhibit insulin signaling pathways. It is unknown however whether the excess ceramide is generated proximal to the insulin receptor, that is, at the plasma membrane (PM), where it could affect the insulin signaling pathway directly, or the onset of insulin resistance is due to ceramide-induced mitochondrial dysfunction and/or lipotoxicity. Using hepatic cell lines and primary cultures, gain- and loss- of function approach, and state-of-the art lipid imaging, this study shows that PM-associated neutral sphingomyelinase 2 (nSMase2) regulates ceramide homeostasis in fat-loaded hepatocytes and drives the onset of insulin resistance. Our results provide evidence of a regulated translocation of nSMase2 to the PM which leads to local generation of ceramide and insulin resistance in cells treated with palmitic acid (PAL), a type of fat commonly found in diabetogenic diets. Oleic acid, which also causes accumulation of lipid droplets, does not induce nSMase2 translocation and insulin resistance. Experiments using the acyl-biotin exchange method to quantify protein palmitoylation show that cellular PAL abundance regulates the rate of nSMase2 palmitoylation. Furthermore, while inhibition of nSMase2 with GW4869 prevents PAL-induced insulin resistance, the overexpression of wild type nSMase2 but not palmitoylation-defective mutant protein potentiates the suppressive effect of PAL on insulin signaling. Overall, this study identifies nSMase2 as a novel component of the mechanism of insulin resistance onset in fat-loaded hepatocytes, that is, cell-autonomous and driven by PAL.

The bioactive lipid ceramide mediates a plethora of cellular stress responses. Numerous diseases have been associated with a shift in ceramide homeostasis, either in the direction of chronic overproduction (metabolic syndrome, Alzheimer’s, atherosclerosis) (1, 2, 3, 4) or deficiency (cancer) (5, 6, 7, 8). Nonalcoholic fatty liver disease (NAFLD) and its complications, nonalcoholic steatohepatitis, and cirrhosis also manifest with an accumulation of distinct ceramide species in the liver. This accumulation of ceramides has been linked to lipotoxicity, insulin resistance (IR), and mitochondrial dysfunction. The exact mechanism by which ceramide exerts these negative effects is not completely understood, but it is thought that ceramide may interfere with insulin signaling pathways in some tissues, particularly in the liver and the muscle. Proving the causative link between ceramide and IR has been challenging because ceramide is a hydrophobic molecule and exerts its biological effects at the specific subcellular site of its generation. Whether NAFLD and diabetes in general are accompanied by an excessive generation of ceramide at the plasma membrane (PM), in a proximity to the insulin receptor, and what are the enzymes that are responsible has never been tested.

In the cells, ceramide generation is strictly compartmentalized. De novo ceramide synthesis occurs in the ER and is initiated by the serine palmitoyltransferase (SPT)-catalyzed formation of a sphingoid base from palmitoyl-CoA and serine. A family of six ceramide synthases (CerS1-6), each with a preference towards fatty acids of particular chain length, acylate the primary amine of the sphingoid base to produce dihydroceramides that are then reduced to ceramides by desaturase 1. Ceramides travel to the Golgi by a vesicular- or protein-dependent transport. At the Golgi, ceramide is converted to more complex sphingolipids (i.e., SM and glycosphingolipids), which are then delivered to the PM via exocytosis/secretory pathways. The de novo pathway of ceramide synthesis has been extensively studied in respect to NAFLD, as palmitic acid (PAL), a 16:0 fatty acid, which is the most detrimental type of fat associated with the progression of NAFLD, is also the rate-limiting and key substrate for SPT. Increased PAL abundance has been known to elevate SPT in hepatocytes (9, 10). Similar activation of SPT was seen in the liver following the consumption of diet rich in saturated fats (11). Inhibitors of SPT, CerS, and Des1, that all suppress sphingolipid synthesis by inhibiting different steps in the de novo pathway, have demonstrated beneficial effects in improving hepatic and nonhepatic manifestations of NAFLD (i.e., the excessive lipid droplets accumulation in steatosis, whole body IR, hyperglycemia, and hypertriglyceridemia) in animal models (12).

The sphingomyelinases are a group of five biochemically and genetically distinct enzymes (SM phosphodiesterase *SMPD1-5*) that govern a second major metabolic pathway for ceramide generation. All sphingomyelinases catalyze the hydrolysis of SM to ceramide and phosphorylcholine but differ in their pH optimum and subcellular location (13). Neutral sphingomyelinase 2 (nSMase2, encoded by the *SMPD*3 gene) is a signaling enzyme, that is activated by cytokines like IL-1β and TNFα, during growth arrest and oxidative stress, as well as in the course of other cellular stress responses (14, 15, 16). Some diets have been shown to influence the activity of nSMase2 through transcriptional effects (17, 18). Downregulation of the *SMPD3* by hypermethylation is associated with the development of hepatocarcinoma (19), renal cell carcinoma (20), mammary (21), squamous cell (22), non-small cell lung (23) carcinomas, while a mutation interfering with its PM localization is found frequently in leukemias (24). Vice versa, *SMPD3* was identified as one of the seven core genes exhibiting reduced promoter methylation in association with diabetic nephropathy (25). Among all genes encoding sphingolipid-metabolizing enzymes, *SMPD3* has shown the highest association coefficient with NAFLD according to the Comparative Toxicogenomics Database gene associations database (26). Up until now, however, only two studies have indicated that nSMase2 might be involved in obesity-related pathologies by showing evidence in the context of adipose inflammation (27, 28).

nSMase2 is a membrane-associated enzyme of 655 amino acids that is found mainly at the Golgi or the PM (29). The protein contains two helical hydrophobic domains that do not span the membrane but attach the enzyme to the inner leaflet of the membrane bilayer, two palmitoylation clusters, and a catalytic C-terminal domain (30). The nSMase2 cellular localization is affected by certain stimuli such as TNFα, PMA, H_2_O_2_, and cell confluence, all of which have been reported to induce nSMase2 translocation from the Golgi compartment to the PM (14, 31). An earlier study had suggested that nSMase2 traffics from the Golgi to the PM as a membrane protein *en route* to the cell surface and then recycles back to the Golgi through the endosomal/recycling compartment (32).

At cellular level, IR is defined as the suppressed activation of the insulin signaling cascade manifested by a muted phosphorylation of Akt upon insulin stimulation. Extrinsic (i.e., pro-inflammatory cytokines) and intrinsic (i.e., oxidative stress, lipotoxicity, ER stress) factors contribute to IR onset in hepatocytes. Accumulation of diacylglycerol or ceramide, resulting from the excess fat supply to the steatotic liver, also negatively affect Akt phosphorylation. In the case of ceramide, the precise mechanisms are still enigmatic. The following likely scenarios have been proposed: (i) ceramide induces ER stress that leads to IR by activating tribbles 3 protein that can inhibit Akt/PKB signaling pathway (33); (ii) ceramide interferes with the formation of the signaling domains on the membrane, the lipid rafts, that mediate insulin receptor clustering (34); or (iii) ceramide induces mitochondrial dysfunction and oxidative stress which also suppresses insulin cascade. Notwithstanding, an enzyme that could generate ceramide in the proximity of the insulin receptor and consequently to suppress directly the INS response has not been yet identified.

This manuscript describes the first evidence that in response to excess supply of the cells with PAL, but not with oleic acid (OLE), a less detrimental unsaturated counterparts, nSMase2 can generate ceramide at the inner leaflet of the PM, which is responsible for the downregulation of insulin-induced Akt phosphorylation. The underlying mechanisms involve palmitoylation-dependent translocation of the enzyme to the PM. Experiments with pharmacological inhibitor or overexpression of WT and palmitoylation-defective mutant of nSMase confirm that nSMase2 translocation to the PM is causatively linked to the onset of IR in vitro. In conclusion, the present work defines nSMase2 as a novel, cell autonomous mechanism for suppression of insulin signaling cascade in the steatotic liver.

## Material and Methods

### Cells and treatments

HepG2 cells (ATTC, Manassas, VA) were maintained in MEM (Invitrogen, Carlsbad, CA) supplemented with 10% FBS and 1% penicillin/ streptomycin (Invitrogen, Carlsbad, CA) in a humidified atmosphere of 95% air and 5% CO_2_ at 37°C. AML-12 cells (a gift from Dr Terry Hinds, University of KY) were maintained in DMEM plus Ham’s F12 (1:1) (Sigma-Aldrich, St. Louis, MO) supplemented with insulin (0.005 mg/ml), transferrin (0.005 mg/ml), selenium (5 ng/ml), and dexamethasone (40 ng/ml) (Sigma-Aldrich, St. Louis, MO). Typically, cells were cultured in 6-well plate until reaching 70% confluency and were then treated with lipid-free BSA (Sigma-Aldrich, St. Louis, MO, Cat# A6003), with different concentrations of PAL (Sigma-Aldrich, St. Louis, MO, Cat# P5585), or with OLE, (Sigma-Aldrich, St. Louis, MO, Cat# O1383, delivered as a complex with BSA (2:1 by mol) for 18 h in serum-depleted media. At the end of 18 h, the cells were treated with insulin, at the doses and times indicated in the figures. When needed, the nSMase2 inhibitor (GW4869; Cayman Chemical Company, Ann Arbor, MI) was added for 30 min prior to and during insulin treatment. The inhibitor was prepared as a 2 mM suspension of GW4869 in DMSO and stored at −80°C while protected from light. To increase solubility prior its addition to the cells, 5 μl of methane sulfonic acid (5% solution in H_2_O) was added per each 50 μl aliquot of the 2 mM stock, vortexed, heated at 37°C for 10–15 min, and diluted with tissue culture medium to final concentration of 20 μM. Solution containing DMSO and methane sulfonic acid at appropriate proportions was used as a vehicle control (35).

For overexpression experiments, cells were seeded in 60-mm dishes (1.4 × 10^6^ cells) in MEM containing 10% FBS and penicillin-streptomycin. Twenty-four hours later, the cells were transfected with 5 μg DNA of pCMV6-Entry vector expressing either WT or mutant form of nSMase2 using lipofectamine 3000 (Thermo Fisher Scientific, Catalog # L3000008). For the modulation of nSMase2 levels in primary hepatocytes, adenovirus expressing FLAG-tagged mouse nSMase2 (Ad-nSMase2) was used as previously described (15). Adenoviruses encoding DNA oligos encoding a GFP-tagged sense-loop-antisense sequence (GCCCTCATCTTCCCATGTTACTTCAAGAGAGTAACATGGGAAGA TGAGGGC) against the rat/mouse NSMase-2 (Ad-sh-nSMase2) and a scrambled sense-loop-antisense RNA sequence (Ad-scr-nSMase-2) were applied as described in an earlier publication (36).

### Isolation of primary hepatocytes

From the mouse: Livers were perfused through a cannulated vena cava with a prewarmed solution of sterile PBS and high glucose DMEM media containing type I collagenase solution (Thermo Fisher Scientific, Catalog # 17100017, 0.5 mg/ml) using peristaltic pump running at 3 ml/min for 10 min for each solution. Hepatocytes were released from excised livers in a 10 cm Petri dish, filtered through a 70 μm cell strainer, and pelleted at 50 *g* for 90 s at 4°C. Viability was determined by trypan blue staining, and cells from preparations with 85% viability or greater were plated in 6-well plates precoated with collagen I (Sigma-Aldrich, Catalog # C8919). Cultures were maintained in high glucose DMEM (Sigma-Aldrich, Catalog # D5648) containing 0.2% FBS and penicillin-streptomycin for 24 h prior treatment. From the rat: Hepatocytes were isolated from ether-anesthetized male Fisher 344 rats by in situ collagenase perfusion and cultured in culture dishes coated with 6.3 mg/ml Matrigel (BD Bioscience, Franklin Lakes, NJ) in Waymouth’s medium supplemented with insulin (0.15 μM) and penicillin/streptomycin (100U/ml) for 5 days at 37°C in 5% CO_2_.

### Western blotting

Cells were harvested in ice-cold PBS, pelleted at 800 *g* (for HepG2 and AML-12 cells) or 50 *g* (for primary hepatocytes), and lysed for 1 h on ice in 1% Triton X-100, 0.2% SDS, 1 mM EDTA, 3.3 mM EGTA 1 mM Na_3_VO_4_, 15 mM NaF, 2 mM Na_4_P_2_O_7_, 0.8 mM β-glycerophosphate, and 10 mM Tris-HCl, pH 7.4. Lysates were centrifuged at 15,000*g* for 10 min at 4°C and the clear supernatant was used as cell extracts. The protein concentration was measured by Lowry assay. Proteins (50 μg/lane) were resolved by 7.5% or 10% SDS-PAGE and transferred to Immobilon-P PVDF membrane by semidry blotting. The membranes were blocked in Tris-buffered saline (TBS, pH 7.4) containing 5% BSA and 0.05% Tween-20. pAkt and total Akt were detected by incubating the membrane with rabbit anti-Phospho-Akt (Ser473) or rabbit anti-Akt antibody (Cell Signaling Technology, Inc, Danvers, MA) at a dilution of 1:1000 in 1% BSA and 0.05% Tween-20 at 4°C overnight followed by anti-rabbit IgG-alkaline phosphatase-conjugated antibody (Sigma-Aldrich, Catalog # A3812) at a dilution of 1:10,000 at room temperature for 1.0 h. Protein antibody interactions were visualized using the ECF kit (Cytiva, Catalog # RPN5785) and ChemiDoc Imager (Bio-Rad Laboratories, Hercules, CA). Pierce Restore Western blotting stripping buffer (Thermo Fisher Scientific, Waltham, MA) was used when needed.

### Lipid isolation and analysis

Cells were grown to subconfluency in 6-well plates and labeled with ^3^H-PAL (American Radiochemical Corporation, St. Louis, MO) for 18 h. The ^3^H-PAL was mixed with cold palmitate and delivered to the cells as a complex with BSA (2:1, by mol) at low (0.1 mM) or high (1 mM) concentrations, while maintaining the same specific labeling (50 μCi/mmol). Following treatment, cells were harvested, lipids were extracted in the presence of cold carriers, and analyzed as described below. Radioactivity from individual bands was quantified by scintillation counting after scraping the silica off the plate. Lipid extracts from cells were prepared by the method of Bligh and Dyer, modified as described previously (37). Lipids were separated by high-performance thin layer chromatography on silica gel 60 plates using chloroform:methanol:triethylamine:2-propanol:0.25% potassium chloride (30:9:18:25:6, by vol.) as a developing solvent. For mass spectrometry analyses, lipids were extracted from 1.5 × 10^6^ cells with 0.75 ml of CH_3_OH:CHCl_3_ (2:1, by vol) and in the presence of an internal standard cocktail (500 pmol of each species dissolved in a final total volume of 10 μl of ethanol) (Sphingolipid Mix II, catalog number LM-6005, Avanti Polar Lipids (Alabaster, AL)). This single-phase mixture was incubated at 48°C overnight in a heating block. After cooling, 75 μl of 1 M KOH in CH3OH was added and, after brief sonication, incubated in a shaking water bath for 2 h at 37°C to cleave potentially interfering glycerophospholipids. The mixture was then neutralized with glacial acetic acid and phases were separated by the addition of 1 ml of CHCl_3_ and 2 ml of H_2_O and centrifugation using a table-top centrifuge. The lower layer (the “organic-phase extract”) was transferred to a new tube and dried using a Savant AES2000 Automatic Environmental Speed Vac. The dried residue was reconstituted in 0.3 ml of the appropriate mobile phase solvent for LC-MS/MS analysis. Ceramides, SM, and hexosyl ceramides were analyzed using normal-phase LC on a Supelco 2.1 (i.d.) × 50 mm LC-NH2 column, followed by mass spectrometry using an ABI 4000 quadrupole-linear ion trap mass spectrometer with internal standards from Avanti Polar Lipids (Alabaster, AL) as described (38).

### Cysteine to alanine site-directed mutagenesis of nSMase-2

The cDNA clone of mouse SM phosphodiesterase 3 (*Smpd3*; NM_021491) in mammalian expression vector (pCMV6-Entry) with C-terminal Myc- DDK Tag was purchased from OriGene Technologies (Catalog # MR209795). The mouse nSMase-2 gene has 22 cysteine residues, with five of these (Cys^53^, Cys^54^, Cys^59^, Cys^395^, and Cys^396^) having previously been shown to undergo palmitoylation (30). These five cysteines were mutated to alanines to produce a complex mutant, named C-A(5)-nSMase2, sequentially using synthetic oligonucleotides and the QuikChange Lightning Site-Directed Mutagenesis Kit (Agilent Technologies, Catalog # 210513). The sequence of the oligonucleotides used for mutagenesis (Integrated DNA Technologies were as follows: For the C53A, C54A mutant: 5′-gcagaacagctgcagggcggcggggtcatctgccctc-3′ and 5′-gagggcagatgaccccgccgc cctgcagctgttctgc-3’. For C59A: 5′-gtgaagaggaccgtggcgaacagctgcaggca-3′ and 5′-tgcctgcagctgttcgccacggtcc tcttcac-3'. For C395A, C396A: 5′-gctgttgagacatttgaaattggcggcaccatgacaaccgtagacccca-3′ and 5′-tggggtcta cggttgtcatggtgccgccaatttcaaatgtctcaacagc-3'.

### S-acyl biotin exchange assay

HepG2 cells transfected with WT nSMase2 or the C-A(5)-nSMase2 mutant were treated with PAL as described, harvested in ice-cold PBS, and pelleted at 800xg. Cell pellets were lysed in 155 μl PBS supplemented with 1.7% Triton-X100, 10 mm N-ethylmaleimide (NEM; Sigma-Aldrich, Catalog # E3876), and protease inhibitor cocktail and incubated at 4°C with continuous rotation for 1 h. The inclusion of NEM allowed for irreversible blockage of all free thiols in the sample. Fifteen microliters of each lysate (referenced to by the letter “c”) was reserved as a total whole cell lysate to check for protein expression before S-acyl biotin exchange (ABE) assay. The remaining 140 μl were subjected to chloroform/methanol precipitation (39). Efficacy of nSMase2 precipitation was verified by the lack of signal in the supernatant following precipitation by Western blotting with anti-FLAG antibody. The pellets were resuspended in solubilization buffer (4% SDS, PBS, 5 mm EDTA, pH 7.4) containing 10 mm NEM. Solubilized proteins were diluted with lysis buffer containing 1.0 mM NEM, 0.2% Triton-X100, and protease inhibitors and incubated overnight at 4°C with rotation. After another chloroform/methanol precipitation, proteins were resuspended in solubilization buffer and divided into two equal parts (referenced to with the letters “b” and “c”); first one was treated with control buffer (PBS, 1.0 mM biotin-BMCC (Thermo Fisher Scientific, Catalog # 21900), 0.2% Triton-X100, pH 7.4) and the second one, with the same buffer containing hydroxylamine (HAM) (0.7 m, Sigma-Aldrich, Catalog # 46780-4) in order to specifically cleave palmitate (or other acyl group) linked via ester bonds to cysteine residues and incubated at room temperature for 1 h. All samples (HAM-treated or not) were further precipitated with chloroform/methanol precipitation, resuspended in solubilization buffer, diluted in low biotin-BMCC buffer (PBS containing 5 mm EDTA, 0.2 mm biotin-BMCC, and 0.2% Triton-X100, pH 7.4), and incubated at room temperature for 1 h with rotation. After that, samples were precipitated once again, resuspended in solubilization buffer containing 0.1% SDS, 0.2% Triton X-100, and protease inhibitors, incubated at room temperature for 30 min (with rotation), centrifuged at 20,000 *g* to remove any insoluble material, and incubated with 15 μl of high-capacity Pierce NeutrAvidin-agarose (Thermo Fisher Scientific, Catalog # 29200) at 4°C overnight to capture biotin-labeled proteins. Beads were washed with lysis buffer containing 0.1% SDS and 0.2% Triton X-100, eluted with 4×SDS sample buffer containing 5 mM DTT, and boiled at 80°C. Samples (the initial 15 μl lysates and the biotinylated proteins from the two 70 μl aliquots) were run on 10% SDS-PAGE and nSMase2 abundance in each sample was analyzed and quantified by Western blotting with primary anti-FLAG antibody (OriGene Technologies, Clone OTI4C5, 1:2000 dilution) and secondary anti-mouse IgG-alkaline phosphatase-conjugated antibody (Sigma-Aldrich, Catalog # A2418) at a dilution of 1:10,000. The rate of palmitoylation was calculated as (A-B)/4.7xC, where A and B are the intensity of FLAG-positive band in samples a and b (that is, in the presence and in the absence of HAM), C is the intensity of the FLAG-positive band in the starting lysates, and 4.7 is a correction factor for differences in volume.

**Fig. 1:**
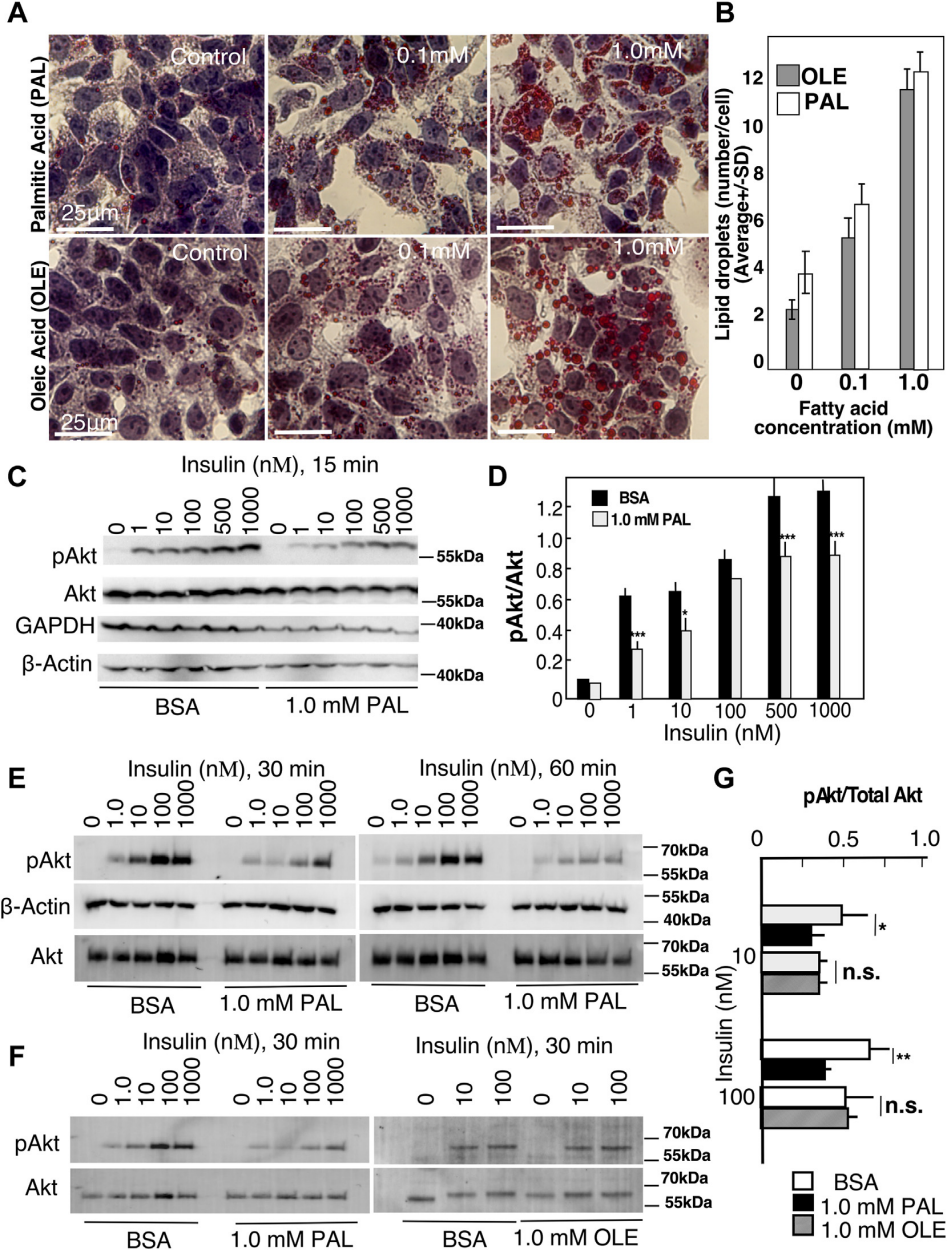
Difference in the onset of steatosis and insulin resistance in HepG2 cells following treatment with free fatty acids. HepG2 cells were treated with oleic acid (OLE) or palmitic acid (PAL), at the indicated concentrations or with vehicles as a control for 18 h. Free fatty acids were delivered as complexes with fatty acid free BSA (BSA: fatty acid, 1:2, by mol.). A: Lipid droplet accumulation visualized with Oil Red O and hematoxylin staining; (B) quantification of the number of lipid droplets per cell. Approximately 300 cells per group were counted. C–G: Western blot analysis of Akt phosphorylation following treatment with insulin at the indicated concentrations and times. Antibodies against pAkt (S473) and total Akt were used to assess Akt activation. β-actin and GAPDH were used as loading controls. Representative western blots (C, E, F) and quantification (D, G) are shown. All experiments were performed several times (between three and five) with cells at different passage number. In each independent experiment, three dishes were used per time/dose point as technical replicates. Data shown are average+SD (n=3) and representative of 3–5 independent experiments. ∗ *P*<0.05, ∗∗, *P*<0.01, ∗∗∗, *P*<0.005.

**Fig. 2:**
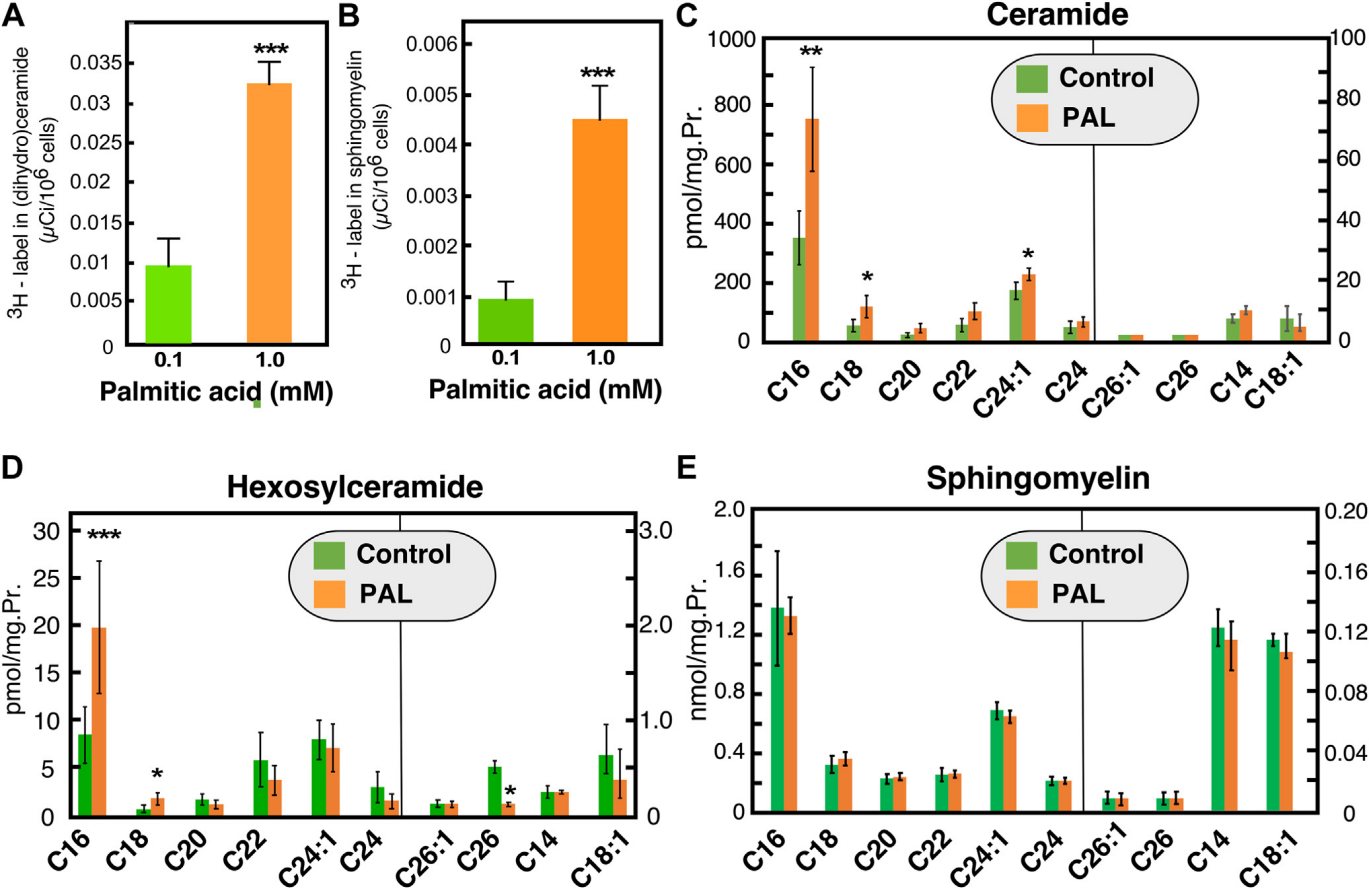
Palmitate-induced changes in sphingolipid metabolism. HepG2 cells were treated with PAL (1 mM, delivered as BSA complex) or with 0.1 mM PAL, as the control. A-B: Flux of palmitate in ceramide and SM synthetic pathways. Cells labeled with [^3^H]-palmitic acid at a constant specific activity of 50 mCi/mmol and supplied to cells at the indicated concentrations for 15 h. Cells were harvested, lipid was extracted in the presence of nonradiolabeled carriers, ceramide, and SM, and analyzed by autoradiography after separation with TLC. Incorporation of the label in ceramide (A) and SM (B) was quantified by scintillation counting after scraping the corresponding spots from the TLC plate. C-E: Effect of PAL treatment on the levels of ceramide (C), hexosylceramide (D), and sphingomyelin (E). Lipids were extracted from the cells and analyzed by mass spectrometry. Data are average+SD, n=3. ∗ *P*<0.05, ∗∗, *P*<0.01, ∗∗∗, *P*<0.005. PAL, palmitic acid.

**Fig. 3:**
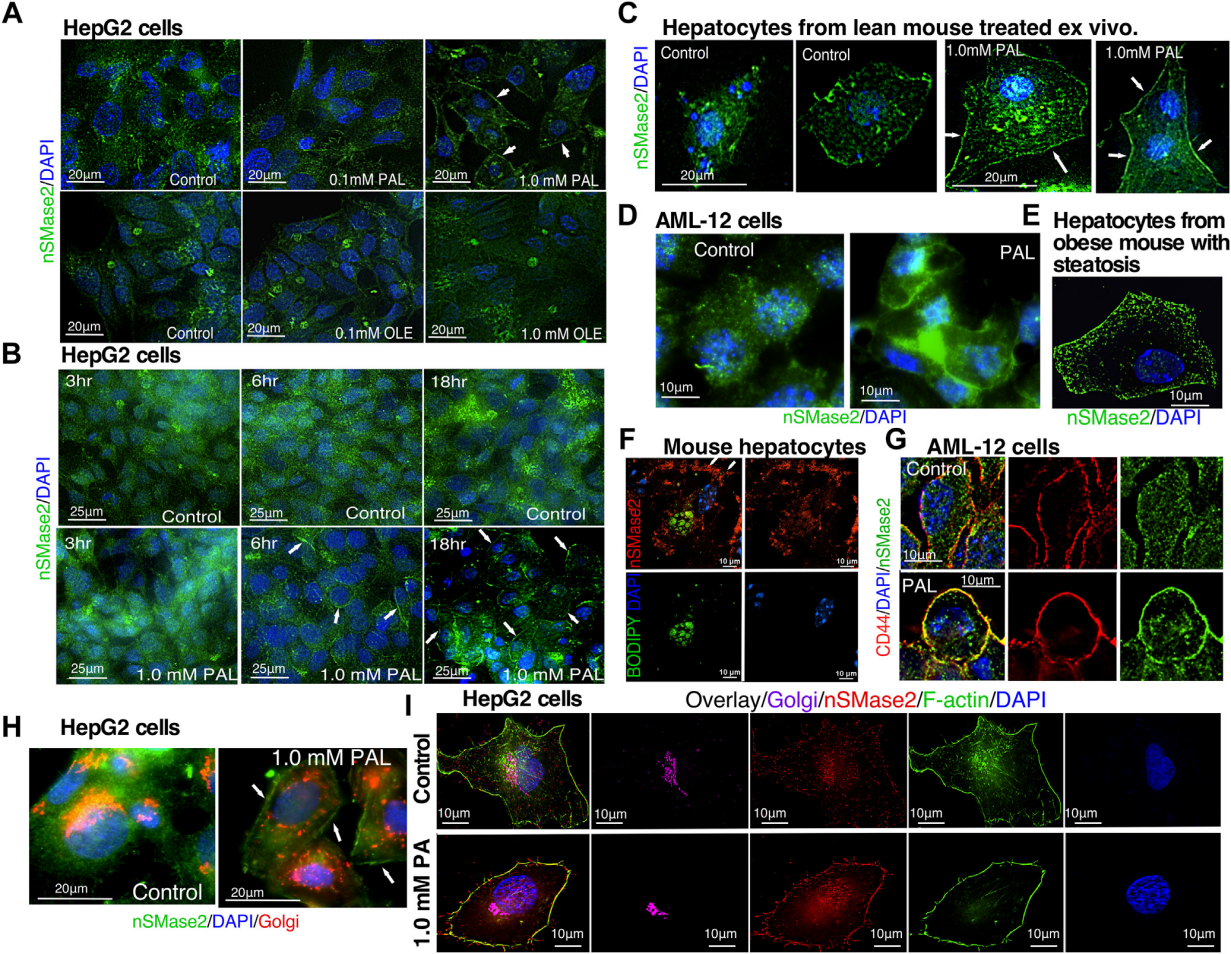
Subcellular localization of nSMase2 in hepatic cell lines or primary hepatocytes following palmitate treatment ex vivo or in hepatocytes from mice with diet-induced obesity. A-F: Palmitate-induced translocation of nSMase2 to the plasma membrane. Various hepatic cell lines and primary hepatocytes were treated with PAL or OLE as indicated. Immunolabeling was done with our custom-made polyclonal anti-nSMase2 antibody (green in A-E, red in F) while nuclei were stained with DAPI (blue). For super resolution microscopy, BODIPI was used to stain lipid droplets (F). A: HepG2 cells treated with PAL or OLE for 18∖xA0h; (B) HepG2 cells treated with PAL for the indicated times; (C) primary mouse hepatocytes treated with PAL ex vivo for 18∖xA0h; (D) AML-12∖xA0cells were treated with PAL for 18∖xA0h; (E, F) primary hepatocytes isolated from mouse with diet-induced steatosis analyzed by traditional confocal microscopy (E) or super-resolution microscopy (SIM) (F). G-I: Effects of PAL on the colocalization of nSMase2 with Golgi and the plasma membrane markers. HepG2 and AML-12∖xA0cells were treated with PAL or vehicles as indicated. G: AML-12∖xA0cells labeled with anti-CD44 antibody (red) or anti-nSMase2 antibody (green) and costained with DAPI (blue). H: HepG2 cells immunolabeled with anti-nSMase2 antibody (green), mouse anti-GM130 antibody (red) and costained with DAPI (blue). I: HepG2 cells immunolabeled with anti-nSMase2 antibody (red), anti-GM130 antibody (purple) and costained for F-actin with Alexa Flour 488 phalloidin (green) and for the nuclei, with DAPI (blue). All pictures shown are representative of multiple independent experiments (n=5 for panel A, 2 for panel B, 2 for panel C, 3 for panel D, 3 for panel F, and 1 for panels G and H). nSMase2, neutral sphingomyelinase 2; PAL, palmitic acid; OLE, oleic acid.

**Fig. 4:**
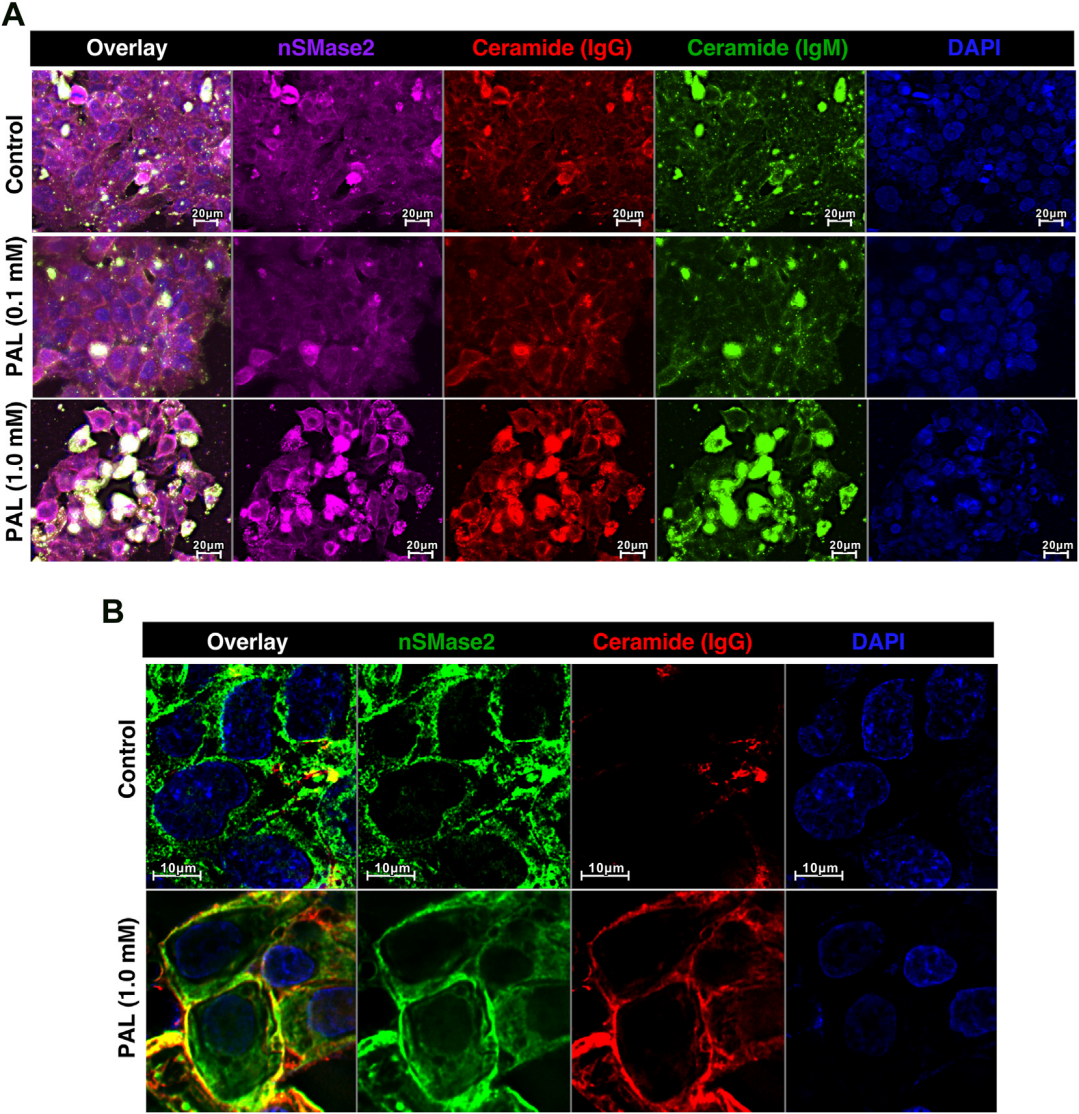
Visualization of ceramide in steatotic HepG2 cells. HepG2 cells treated with indicated concentrations of PAL or vehicle control for 18 h. Immunolabeling of fixed cells was done with anti-ceramide IgG (red), anti-ceramide IgM (green), or anti-nSMase2 IgG (magenta). Nuclei were staining with DAPI (blue). Images were acquired at 20x (A) or 60x (B) magnification. Arrows point to the PM. PM, plasma membrane; nSMase2, neutral sphingomyelinase 2; PAL, palmitic acid.

### Oil red O staining

HepG2 cells were grown on a coverslip (VWR International, Catalog # 48366089) at an initial density of 1x10^6^ cells/well in a 6-well plate (Falcon, Catalog # 353224). Upon reaching 70% confluency, the cells were treated with different concentrations of PAL or OLE, delivered as complex with fatty acid-free BSA (2:1, by mol) for 18 h. Cells were then washed three times with ice-cold PBS and fixed with 4% paraformaldehyde (Thermo Fisher Scientific, Catalog # J19943K2) for 30 min. After fixation, cells were washed three times and stained with oil red o (ORO)-isopropanol solution (Electron Microscopy Sciences, Catalog # 2607905) at 6:4 ratio with dH_2_O and let to stand for 10 min at room temperature. Cells were washed again with PBS to remove unbound staining. Then, cells were counterstained with Hematoxylin GiIl 2X (VWR International, Catalog # 10143146) for 30 s and coverslips were mounted on a glass slides using Epredia aquamount mounting medium (Thermo Fisher Scientific, Catalog #143905). Lipid droplets were then visualized under the Nikon Eclipse Ti2 microscope. Lipid droplet numbers and nuclei (cells) numbers were analyzed by ImageJ (https://imagej.net/ij/index.html) software. At least 3 random fields (∼300 cells total) from each treatment condition were analyzed. Then, the average number of lipid droplets per nucleus was calculated.

### Indirect immunofluorescence

Transfected and nontransfected HepG2 cells, AML-12 cells, or primary hepatocytes cultured on collagen-covered coverslips were washed three times with PBS containing 5 mM MgCl_2_ and fixed with 4% paraformaldehyde for 30 min at room temperature. Coverslips were washed again with MgCl_2_, treated with 10 mM NH_4_Cl (5 min), and permeabilized with 0.2% Triton X-100 for 15 min on ice. After blocking with 1% BSA in PBS-MgCl2 for 1 h at room temperature, the following primary antibodies were used: custom-made rabbit nSMase-2 (1:200 dilution), mouse anti-FLAG antibody (OriGene Technologies, Clone OTI4C5, 1:1000 dilution), rabbit anti FLAG (Sigma-Aldrich, Catalog # F7425, 1:150 dilution), mouse anti-GM130 antibody (a marker for Golgi apparatus and gift from Dr Erhard Bieberich (University of Kentucky, Lexington, KY)), and mouse LAMP1 antibody (Santa Cruz, clone H4A3, 1:100). After washing with PBS-MgCl2-BSA, cells were incubated for 1 h with a secondary antibody of the appropriate species: Alexa 488 goat anti-rabbit (Thermo Fisher Scientific, 1:500), Alexa 568 donkey anti-mouse (Thermo Fisher Scientific, 1:500), and Cy3 or Cy5 anti-mouse (Jackson laboratories, 1:300). Co-staining with 493/503 BODIPY (1 μg/ml) was done according to manufacturer conditions to visualize lipid droplets. Cortical F-actin staining was used as a marker for the PM using Alexa Flour 488 phalloidin (Molecular Probes) as recommended by the manufacturer. Rat anti-mouse CD44 primary monoclonal antibody (cat# 550538, 1:50, BD biosciences, Franklin Lakes, NJ) and Cy3 AffiniPure goat anti-rat IgG secondary antibody (cat# 112-165-062, 1:100 dilution, Jackson ImmunoResearch Inc., West Grove, PA) were also used to stain the PM. The coverslips were washed three more times in PBS-MgCl_2_-BSA and mounted with Fluoroshield mounting medium containing 4, 6-diamidino-2-phenylindole (Sigma-Aldrich, Catalog # F6182). Cell staining was analyzed on Nikon Eclipse Ti2 microscope.

The anti-nSMase2 antibody was raised against a recombinant 437 amino-acids in length fragment of the murine nSMase2 (^195^Gly-^432^Phe) as an antigen. The fragment was first fused to a C-terminal Trx/His and an N-terminal His tag using the PEt-32a plasmid to increase solubilization and allow purification. The fusion protein was expressed in the BL21(DE3) RP cells and purified using a large scale His-Bind Kit (Novagen, Morgantown, WV) which also involved the removal of the N-terminal tags by enterokinase cleavage. Polyclonal antibodies were generated using a two-rabbit protocol and affinity purified against the antigen at BioSource, Inc. (Hopkinton, MA).

### nSMase2 and ceramide colocalization

Cells or liver tissue were fixed with 4% paraformaldehyde. Samples were subjected to mild permeabilization with 0.2% Triton X-100/PBS for 10 min at RT, which was followed by blocking any nonspecific-binding sites with 3% ovalbumin/PBS for 60 min at 37°C. Sections were incubated overnight at 4°C with the primary antibody combination: anti-ceramide rabbit IgG (1:100) (40), anti-ceramide mouse IgM MAB0014 (Glycobiotech, 1:50), and anti-nSMase2 mouse IgG (Santa Cruz, sc-166637, 1:100) and with our custom-made rabbit anti-nSMase2 (1:200). After washing with PBS, sections were incubated for 2 h at 37°C with fluorescent secondary antibodies, washed again, and then embedded in Immumount (Sigma). Z-stacked images were taken with a Nikon Eclipse Ti2 fluorescence microscope and then processed using Nikon NIS-Elements software (https://www.microscope.healthcare. nikon.com/products/software/nis-elements) with a 3D deconvolution program.

### NSMase activity assay

Cells from each dish were harvested, resuspended in 1 mM EDTA, 1 mM Na_2_VO_4_, 1 mM NaF, 1:200 (v/v) protease inhibitor cocktail, 10 mM Tris-HCl, pH 7.4), incubated on ice for 30 min, lysed with three consecutive freeze-thaw cycles, and homogenized by sonication for 5 min. NSMase activity was determined as described previously using C6-NBD-SM as a substrate (41, 42). A total of 50 μg of protein was incubated in an assay mix (50 mM Tris-HCl, pH 7.4, 20 μM NBD-SM, 7.5 mM MgCl_2_, 6.6 mM NaF, and protease inhibitors) for 10–60 min at 37°C under shaking. The reaction was stopped by adding 1 ml of methanol. Cell debris was pelleted by centrifugation for 10 min at 16,000 *g*. The supernatants were analyzed by HPLC on a reverse-phase column (Nova PAC, C18) using methanol-water-phosphoric acid (950:50:0.150, v/v) as a mobile phase at a flow rate of 2 ml/min.

### Animals

To obtain primary hepatocytes from obese or lean mice, male C57Bl6 mice were placed on Teklad TD06414 diet (60:21:18 (% cal) fat: carbohydrates: protein). Mice were on this diet for 12–16 weeks. All animal protocols were approved by the Animal Care and Use Committee of University of Kentucky. All mice were housed in controlled environment with 12-h light/12-h dark cycle.

### Statistical analyses

Analyses were conducted using three dishes as replicates per experimental point. After quantification, data were calculated as average ± SD. Statistical significance of differences was assessed using Student’s *t* test or two-way ANOVA. All graphs present the average, SD, and statistical significance of changes calculated for the three replicates in an individual experiment. Each experiment was repeated at least two times (some three or four times) using cells at different passage number.

## Results

### Palmitate- but not oleate-induced lipid droplet accumulation is associated with suppressed insulin response

**Supplemental Fig. 1:**
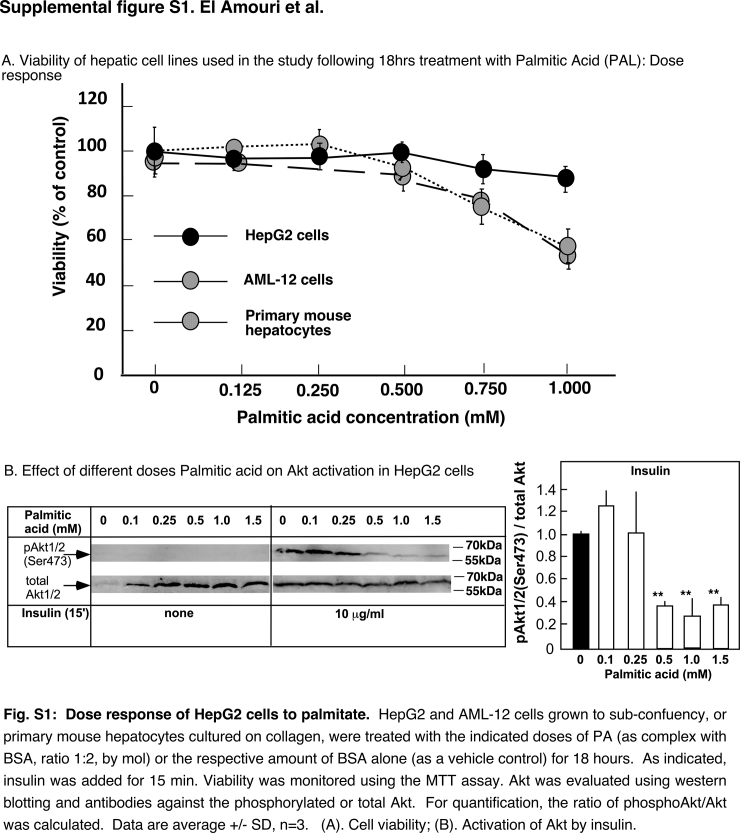


To characterize HepG2 cells as an in vitro model of steatosis and IR, C16:0 PAL or C18:1 OLE was added to the cells as a complex with fatty acids-free BSA for 18 h. Labeling with ORO showed that both, the PAL and OLE treatments, caused lipid droplet accumulation to a similar degree (Fig. 1A, B). ORO-positive droplets were scarcely observable and small in size at the low, 0.1 mM, concentration but abundantly present and larger in size at 1 mM PAL and OLE (Fig. 1A). There were no differences in lipid droplet number per cell between PAL- and OLE-treated cells (Fig. 1B). To test whether lipid droplet accumulation was associated with IR, cells were treated with insulin for 15, 30, and 60 min at doses ranging from 1 to 1000 nM (Fig. 1C–E), and Akt phosphorylation was assessed by Western blotting. In control cells, insulin induced a dose-dependent phosphorylation of Akt as early as 15 min; this induction, however, was inhibited in cells treated with PAL. Similar differences between control and PAL-treated cells were seen following 30 and 60 min of insulin stimulation (Fig. 1E). In contrast, cells that were treated with OLE (Fig. 1F, G) retained normal response to insulin, in spite that these cells exhibited a similar degree of lipid droplets accumulation. These results show that, at least in vitro, lipid droplet accumulation due to excess supply with palmitate, but not oleate, was associated with IR. These effects were seemingly independent of any PAL-associated lipotoxicity (supplemental Fig. S1A). Studies assessing the dose response of HepG2 cells to PAL treatment (i.e., 0. 0.125, 0.25, 0.75, 1.0 mM) showed only insignificant toxicity, (approx. 15%) at the highest 1.0 mM PAL, based on MTT assay. In contrast, the insulin-induced Akt phosphorylation was suppressed by PAL at significantly lower doses (supplemental Fig. S1B).

### Palmitate treatment is associated with changes in sphingolipid metabolism

One of the proposed mechanisms of the detrimental effects of PAL in hepatocytes is via its effects on ceramide homeostasis. Indeed, labeling with ^3^H-palmitate showed that 1 mM PAL increased the partitioning of the labeled precursor into ceramide (Fig. 2A) and SM (Fig. 2B), an indicative for stimulation of the de novo pathway for sphingolipid synthesis. This agrees with previously published data (10, 43). Mass spectrometry-based analyses revealed that the levels of C16:0- and C18:0-ceramide increased more than two-fold following PAL treatment. C24:1-ceramide also increased, albeit at a smaller extent (Fig. 2C). Hexosylceramide (glucosyl- and galactosyl-) levels also increased (Fig. 2D), indicating that the excess palmitate had broad effect of stimulating the de novo synthesis of ceramide and complex sphingolipids. However, despite the 4-fold increase in the ^3^H-PAL incorporation into SM (Fig. 2B), there was no net increase in SM mass (Fig. 2E). One of the several possible explanations for this is that palmitate also induces a hydrolysis of SM from a pool that is difficult to label.

### Characterization of a custom-made antibody against nSMase2

**Supplemental Fig. 2:**
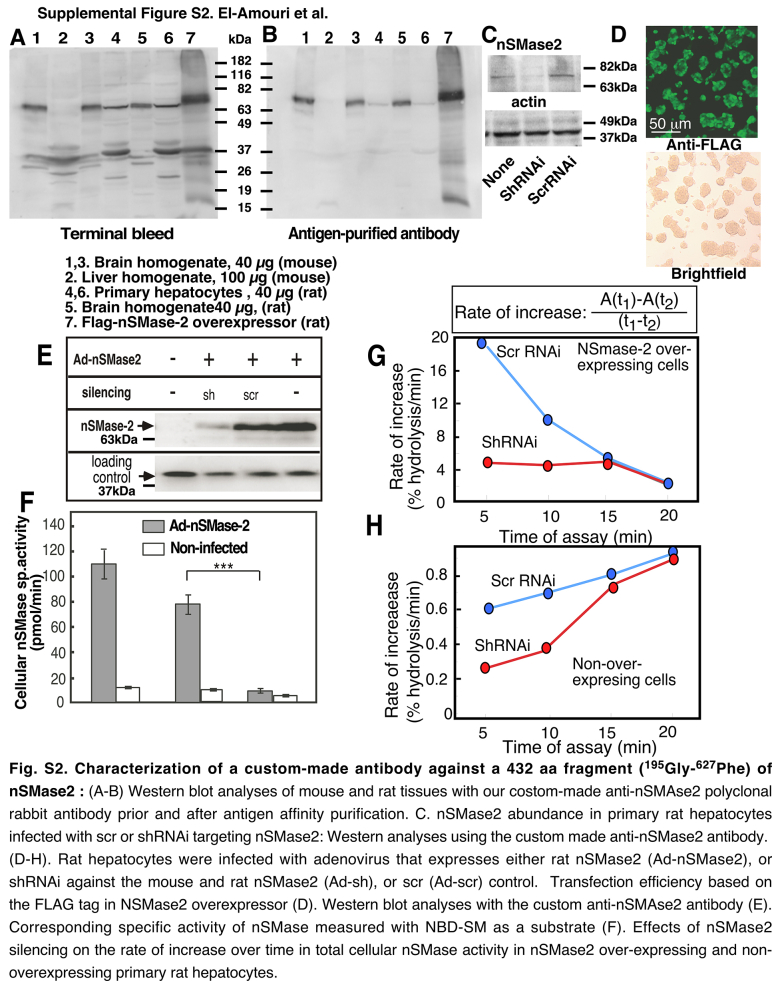


To investigate the latter option, we focused on nSMase2 and made and characterized a novel antibody against a fragment of nSMase2, representing residues 195–627 of the 655-amino acids in length protein. On Western, the antigen-purified antibody recognized a single band with the anticipated molecular weight of 72–75 kDa in several tissues (supplemental Fig. S2A, B). The band was highly abundant in the brain but had low intensity in the liver. Such low abundance of nSMase2 in the healthy liver has been previously seen (18) and corresponds well to data in expression databases (44). To confirm that the weak band seen in liver extracts is indeed nSMase2, we employed an shRNAi approach. Primary rat hepatocytes were treated with adenovirus expressing GFP-tagged shRNAi against nSMase2 (Ad-sh) or scr control (Ad-scr) which infected almost 100% of the cells, based on GFP fluorescence (supplemental Fig. S2D). The intensity of the putative nSMase2 band has significantly suppressed in Ad-sh–treated cells (supplemental Fig. S2C). To avoid any further doubt stemming from the relatively low abundance of nSMase2 in hepatocytes, additional experiments were done using a double transfection system, in which hepatocytes were infected with the same Ad-sh or Ad-scr but also in the presence of adenoviral construct overexpressing the nSMase2. A strong band of the expected molecular weight was seen in the Ad-nSMase2- and Ad-nSMase2/Ad-scr-expressing cells, but not in the Ad-nSMase2/Ad-sh-infected cells, confirming that the band represents nSMase2 (supplemental Fig. S2E). Lastly, we also measured the NSMase activity in these cells using NBD-SM as an exogenous substrate. In the double-transfecting system, changes in activity strictly correlated with changes in nSMase2 protein abundance (supplemental Fig. S2F). The effects of nSMase2 silencing on the endogenous nSMase activity were again not clearly seen. This is because the assay is not specific for nSMase2 and represents the sum of activities of all Mg^2+^-dependent nSMases: as nSMase2 has low abundance, the assays likely reflect mostly the activity of nSMase1, a major form with yet unknown function found in the ER (18). However, calculations of the “rate of increase” in NBD-SM-hydrolyzing activity at very short times of incubation, that is, between 5 and 15 min, allowed to distinguish a component of the total activity that was sensitive to nSMase2 silencing, thereby confirming independently the efficacy of nSMase2 silencing (supplemental Fig. S2G, H). Together, these analyses show that our new custom-made antibody effectively and specifically recognized nSMase-2 in hepatocytes as a single band on Western. It should be noted that the antibody has been validated also for indirect immunofluorescence using primary hepatocytes from a newly developed nSMase2 KO mouse (data not shown) as part of an ongoing metabolic study, the results of which are currently being prepared for a publication.

### Palmitate induces the translocation of nSMase2 to the PM

**Supplemental Fig. 3:**
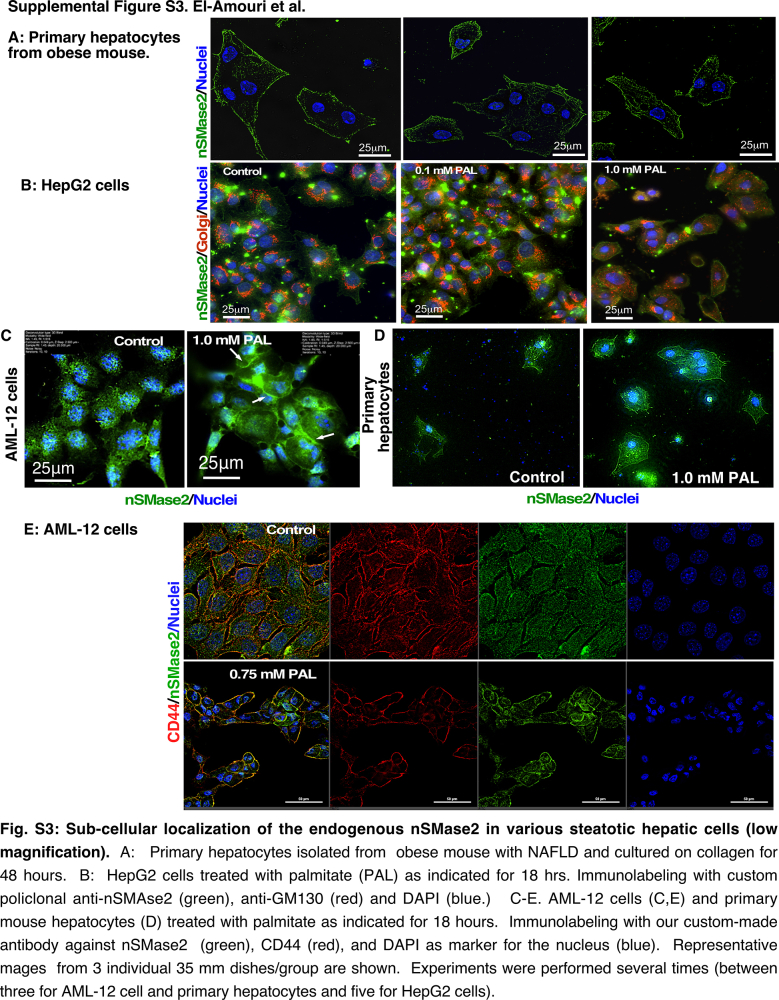


To evaluate the effects of PAL supplementation on nSMase2, we examined first nSMase2 activity, mRNA, and protein levels by the rate of increase in specific nSMase activity, RT-PCR, and Western blotting, respectively. None of these analyses revealed differences between vehicle- and PAL-treated cells (data not shown). Indirect immunofluorescence studies however revealed significant change in nSMase2 subcellular localization. In control HepG2 cells, nSMase2 was seen predominantly in the cytoplasm following a punctuated pattern (Fig. 3A). In PAL-treated cells, the cytosolic fluorescence seemed to decrease and nSMase2 became distinctly visible on the PM (Fig. 3A, B white arrows). In contrast, OLE treatment had no effect on nSMase2 cellular distribution (Fig. 3A, lower panel). This apparent “translocation” of nSMase2 happened as early as 6 h following PAL treatment (Fig. 3B) and was similarly seen in primary mouse hepatocytes (Fig. 3C) and in AML-12 cells, a normal, mouse-derived hepatic cell line (Fig. 3D) (See supplemental Fig. S3 for pictures taken at lower magnification). It should be noted that, as compared to AML-12 and HepG2 cells, in primary hepatocytes, a greater proportion of nSMase2 exhibited PM localization even under control conditions. As primary hepatocytes do not proliferate in culture, such divergence from the rapidly dividing cell lines is to be expected since growth arrest is one of the factor influencing PM localization of nSMase2 (14). Nevertheless, even in primary hepatocytes, PAL-induced translocation of the enzyme was clearly seen.

Colabeling with the Golgi marker, GM130, showed that in control cells, nSMase2 exhibits some Golgi localization (Fig. 3H) which was absent in PAL-treated cells, indicating that most likely, nSMase2 undergoes the Golgi-PM shuttling described earlier in the literature (32). Costaining for GM130, F-actin, and nSMase2 further confirmed that the effects are indeed caused by translocation of nSMase2 form the Golgi to the PM: In control cells, the predominant site for colocalization (indicated in yellow) was between the GM130 and nSMase2 (upper leftmost panel, Fig. 3I), while in the PAL-treated cells, a colocalization was seen almost only between F-actin and nSMase2 (lower leftmost panel, Fig. 3I). The same trend was seen in the overexpression system shown later, where the extent of colocalization was also quantitatively analyzed (supplemental Fig. S5). In addition to F-actin, CD44 was also monitored as a PM marker. Costaining of AML-12 cells confirmed the observations from HepG2 cells, namely that in the PAL-treated cells, nSMase2 localized significantly to the PM (Fig. 3G for single cells and supplemental Fig. S3 for lower magnification), based on the clear overlap between the CD44 and nSMase2 signal.

To test whether localization of nSMase2 to the PM of steatotic cells is also seen in a physiologically relevant system, C57Bl6 mice were placed on a diet enriched in saturated fat for 12 weeks to induce obesity and hepatic steatosis. Primary hepatocytes were then isolated and immunolabeled with antibodies against nSMase2 (Fig. 3E) and costained with BODIPY, a fluorescent label for lipid droplets (Fig. 3F). In the latter case, the immunofluorescence was examined by super-resolution (SIM) microscopy. In both experiments, clear staining of the PM was seen with the anti-nSMase2 antibody, confirming that in hepatocytes from a mouse with diet-induced steatosis, nSMase2 exhibits strong PM localization.

### nSMase2 translocation is associated with elevated ceramide production at the PM

Most of the SM is found in the outer leaflet of the PM, while nSMase2 is tethered to the inner leaflet, and its catalytic site interacts with the cytosolic face of the PM. Whether the inner PM leaflet contains sufficient SM to serve as a substrate for nSMase2 is unclear. SM does not undergo a spontaneous flip-flop across the membrane (45). Only recently, a peripheral myelin protein 2 was found to facilitate trans bilayer movement of SM (46) in model membranes and in MCDK cells but has not been studied in hepatocytes. Therefore, it is important to investigate whether, when translocated to the PM, nSMase2 has access to its substrate SM for local generation of ceramide. To that extent, an immunolabeling with antibodies against nSMase2 and ceramide was done. One of the anticeramide antibodies was developed and characterized in the lab of our collaborator, Dr Bieberich, and was shown to specifically detect ceramide species with different fatty acid chain lengths that include C2, C8, C16, C18, C20, and C24 in various subcellular locations, including the PM (40). As an orthogonal approach, a commercially available anticeramide mouse IgM was used. These experiments revealed a clear colocalization between nSMase2 and ceramide at the PM in HepG2 cells treated with 1 mM PAL. In contrast, cells treated with 0.1 mM PAL or vehicle alone exhibited weak and diffused ceramide positivity (Fig. 4A). Examination at a higher magnification (Fig. 4B) confirmed that the most prominent colocalization between the enzyme and its product was seen at the level of the PM (Fig. 4B), confirming that the nSMase2 PM translocation was paralleled by the local generation of ceramide.

### nSMase2 palmitoylation depends upon palmitate abundance and drives nSMase2 translocation to the PM

**Fig. 5:**
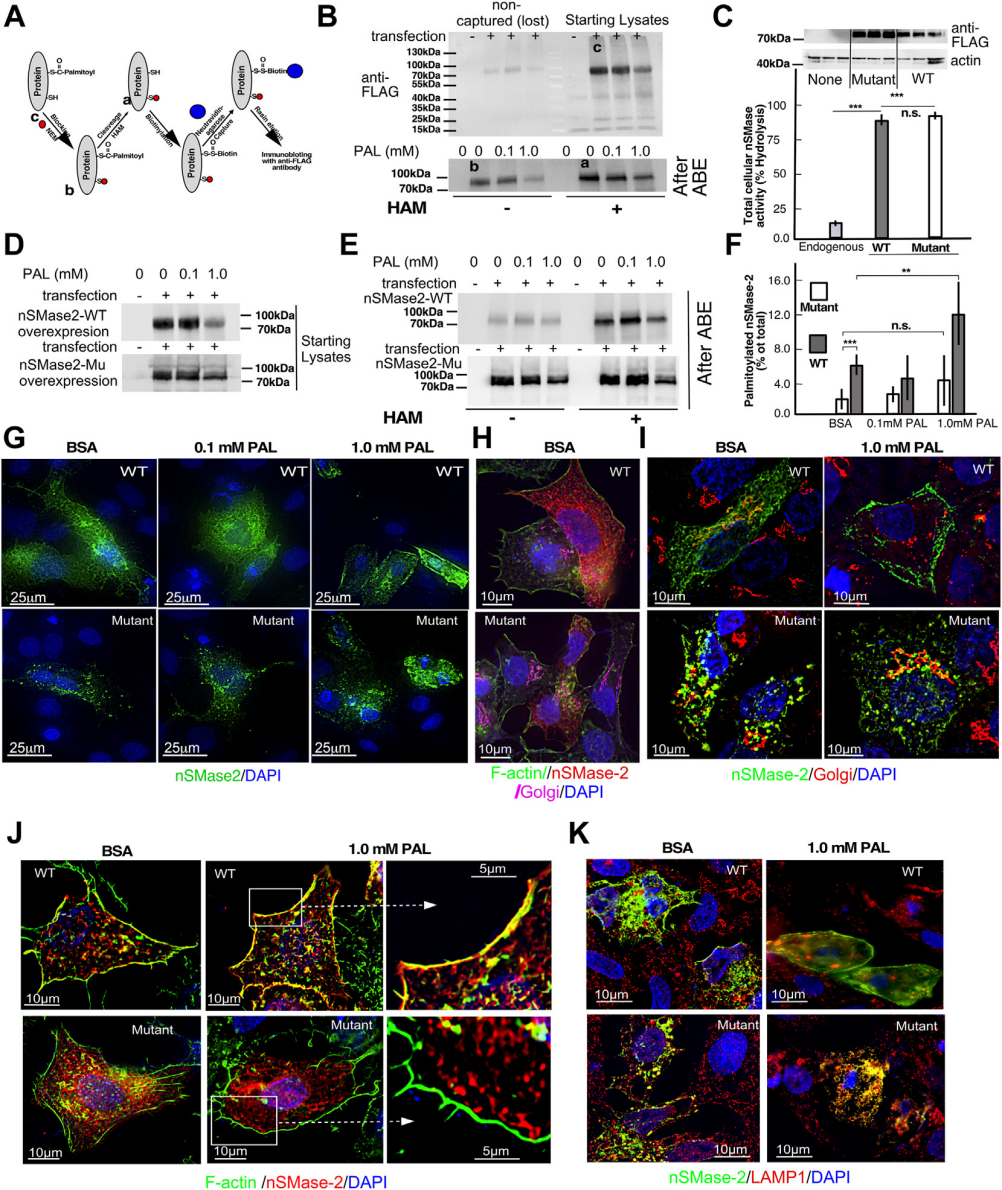
Palmitate treatment increases palmitoylation of nSMase2 and drives PM translocation. HepG2 cells were transfected with FLAG-tagged WT or mutant (^53,54,59,395,396^Cys-to-Ala)-nSMase2 and then treated with PAL at the indicated concentrations for 18 h. A-F: Acyl-biotin exchange (ABE)-based quantification of NSMase2 palmitoylation. A: Schema illustrating the ABE method for quantification of nSMase2 palmitoylation. The letters a, b, and c correspond to the stages in the procedure at which aliquots were analyzed by western and used to quantify the percent palmitoylation according to the formula: (A–B)/(Cx4.7) x100. B: Western blot with anti-FLAG antibody of the starting lysates and the biotin-captured nSMase2 in the absence and presence of HAM. Supernatant after the first precipitation was used as a control for efficacy of precipitation. C: HepG2 cells overexpressing WT or mutant nSMase2 probed with anti-FLAG antibody. Corresponding nSMase activity assay with NBD-SM as an exogenous substrate and HPLC-based separation of the product, NBD-ceramide. D: Total lysates of HepG2 cells overexpressing WT or mutant nSMase2, treated with PAL as indicated, and probed with anti-FLAG antibody. E: Western blot using anti-FLAG antibody of the starting lysates and the biotin-captured nSMase2 in the absence and presence of HAM in HepG2 cells overexpressing WT and mutant nSMase2. F: Palmitoylated nSMase2, calculated as the difference in biotin-accessible nSMase2 in the presence and absence of HAM and normalized for the total nSMase2 (%). G-K: Comparison of PAL-induced PM translocation of WT-nSMase2 and composite nSMase2 mutant (^53,54,59,395,396^Cys-to-Ala). G: Immunolabeling with anti-FLAG antibodies and DAPI of HepG2 cells transfected with WT and mutant nSMase2 and treated with PAL as indicated; (H) immunolabeling of control HepG2 cells expressing WT and mutant NSMase with anti-FLAG (red) and anti-GM130 (purple) antibodies and costating for F-actin (green) and nuclei (blue). I: Immunolabeling of WT and mutant-overexpressing HepG2 cells with anti-FLAG antibody (green), and anti-GM130 antibody (red), and DAPI (blue); (J) immunolabeling of WT and mutant-overexpressing HepG2 cells treated or not with PAL with anti-FLAG antibody (red) with costaining for F-actin with Alexa Flour 488 phalloidin (green) and for the nuclei with DAPI (blue); (K) co-staining for WT and mutant nSMase2 (anti-FLAG, green) and lysosomes (anti-LAMP1, red). DAPI (blue) was used to label nuclei. PM, plasma membrane; nSMase2, neutral sphingomyelinase 2; PAL, palmitic acid; HAM, hydroxylamine.

nSMase2 has been shown to undergo palmitoylation at five Cys residues organized in two clusters, ^53,54,59^Cys and ^395,396^Cys (Fig. 5A) (47). To what extent, if any, the abundance of PAL affects protein palmitoylation in general is not clear and likely depends on the type of protein, the type of cells, and the palmitoyl transferase(s) that are involved. Evidence shows that PAL levels correlate with palmitoylation rates of just a handful of proteins (48, 49, 50). To test this concept in the case of nSMase2, nSMase2 palmitoylation was evaluated in HepG2 cells overexpressing the enzyme using ABE method (Fig. 5A). This method involves irreversible blockade of unmodified SH groups in the protein with NEM, followed by immunoprecipitation and splitting the sample in two halves, one for control treatment and one for treatment with HAM that cleaves acyl groups specifically bound to Cys residues. This is followed by biotin binding of the newly formed reduced SH groups and pull down with Neutravidin. Western blot analyses of entire aliquots taken from the starting precipitate (labeled with C) and those that were treated with HAM (labeled with an A) or with vehicles (labeled with a B) were analyzed by western with anti-FLAG or our custom-made anti-nSMase2 antibody (Fig. 5B–E). The extent of nSMase2 palmitoylation was then quantified according to the formula (A-B)/Cx4.7, where A, B, and C are the intensity of the nSMase2-positive bands in samples from, respectively, HAM-treated, control-treated, and the starting lysate, while 4.7 is a correction factor for volume (51). This approach (e.g., pulldown with Neutravidin and detection with anti-FLAG antibodies) provided more reproducible results than an alternative approach that was first tested (e.g., pull-down with anti-FLAG and immunoblotting for biotinylation). Western blotting of the initial precipitate showed somewhat reduced nSMase2 abundance in PAL-treated cells, which was not due to losses during precipitation (Fig. 5B), but possibly represents lower cell number due to PAL toxicity that ostensibly could be exacerbated by the nSMase2 overexpression. Comparison of the HAM-dependent nSMase2 abundance after ABE showed that indeed, the nSMase2 palmitoylation increased from 4% to 12% following PAL treatment.

Next, we tested the involvement of the two cysteine clusters in this PAL-induced change in nSMase2 palmitoylation and subcellular translocation. A composite mutant C-A(5)-nSMase2 was made by replacing all five Cys with Ala. Mutant and WT nSMase2 were expressed in HepG2 cells, and their capacity for palmitoylation and translocation was compared. Similar levels of overexpression of the WT and C-A(5)-nSMase2 were first confirmed by Western blotting (Fig. 5C, D). To ensure that the C-A(5) mutant was properly folded, SM hydrolyzing capacity of HepG2 cells overexpressing the WT and the C-5(A)-mutant was compared using NBD-SM as an exogenous substrate and HPLC-based separation of the product, NBD-Ceramide. The mutant nSMase2 seemingly retained full functionality, as the increases in nSMase activity seen in HepG2 cells overexpressing the mutant and the WT nSMase2, as compared to nontransfected cells (Fig. 5C), were similar. The decline in nSMase2 abundance following PAL treatment noticeable in the WT-overexpressing cells was not seen in the mutant-overexpressing cells. This might indicate a role of nSMase2 in palmitate-induced toxicity but is beyond the scope of the current study. Unexpectedly, the basal levels of biotinylation of the mutant (e.g., in the absence of HAM) were higher than that of the WT. There are several possible explanations for this. For one, some of the remaining 17 cysteines in the mutant might be resistant to NEM blockage (due to changes in mutant folding, redox state, pH at specific subcellular location, etc.), thereby becoming available to consequent biotinylation. Alternatively, possible failure of the mutant to form dimers, which is mediated by ^561^Cys and ^617^Cys (52), could make additional cysteines available for biotinylation. Nonetheless, this uncertainty regarding the cause for the elevated basal biotinylation of the mutant, in all three groups, the treatment with HAM, had little additional effect. More importantly, the extent of biotinylation observed in PAL (1 mM)-, PAL (0.1 mM)-, and vehicle-treated cells was similar across the board, irrespective of PAL concentration (Fig. 5F). Together, these data indicate that an increased acylation/palmitoylation of nSMase2 is seen in response to PAL and it is likely mediated via the two Cys clusters (^53,54,59^Cys and ^395,396^Cys) identified earlier.

**Supplemental Fig. 4:**
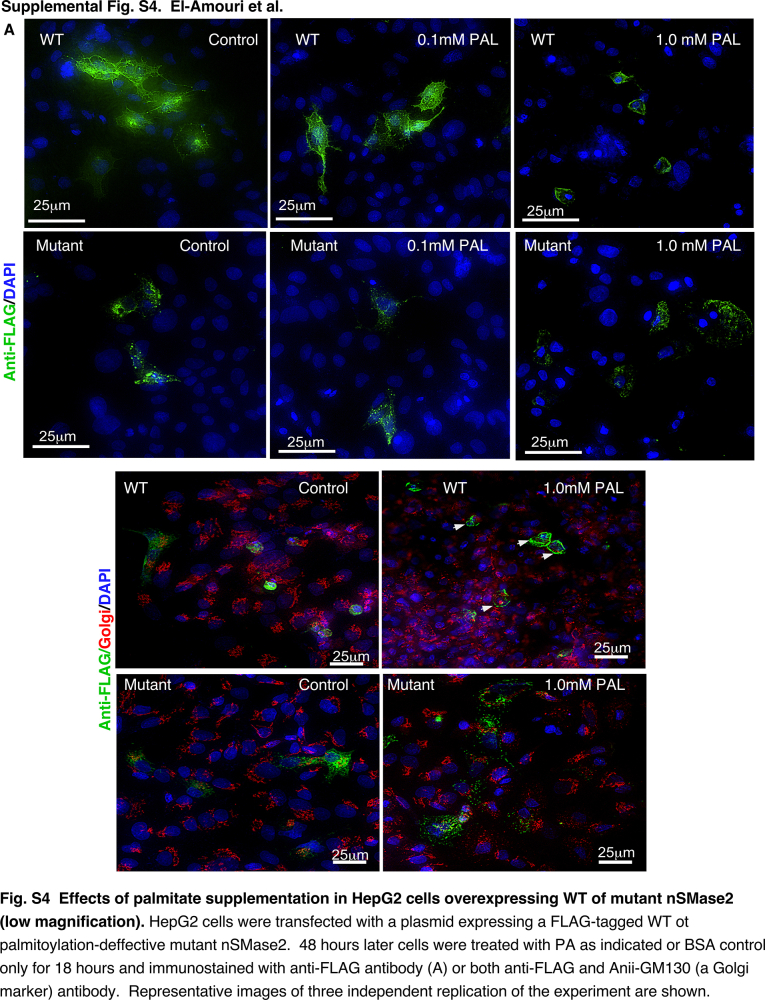


To test whether the increased palmitoylation is driving nSMase2 PM translocation following PAL, immunolabeling studies were done using antibodies against the C-terminal FLAG tag in WT and mutant nSMase2, as well as GM-130, F-actin, and LAMP1 as markers for the Golgi, the PM, and the lysosomes. (Fig. 5G–K, supplemental Figs. S4 and S5). Seemingly, the mutant maintained partial Golgi localization in both, nonstimulated and PAL-stimulated cells like the WT (Fig. 5H, I). In contrast to WT, however, there was no colocalization between the mutant and F-actin following PAL stimulation (Fig. 5J). The extent of colocalization, or the lack of thereof, was quantified and expressed as Pearson and Mander’s test for overlap. Both quantitative tests indicated strong correlation and overlap for WT (supplemental Fig. S5) but none for the mutant (data not shown). Instead, the mutant appears to target the lysosomes, based on a costaining with the lysosomal marker LAMP1 (Fig. 5F). Together, these results show that an increased palmitoylation is responsible for the PM translocation of nSMase2 during steatosis and, at least in part, the abundance of PAL influences nSMase2 palmitoylation rates.

### nSMase2 contributes to IR in steatotic hepatocytes

**Fig. 6:**
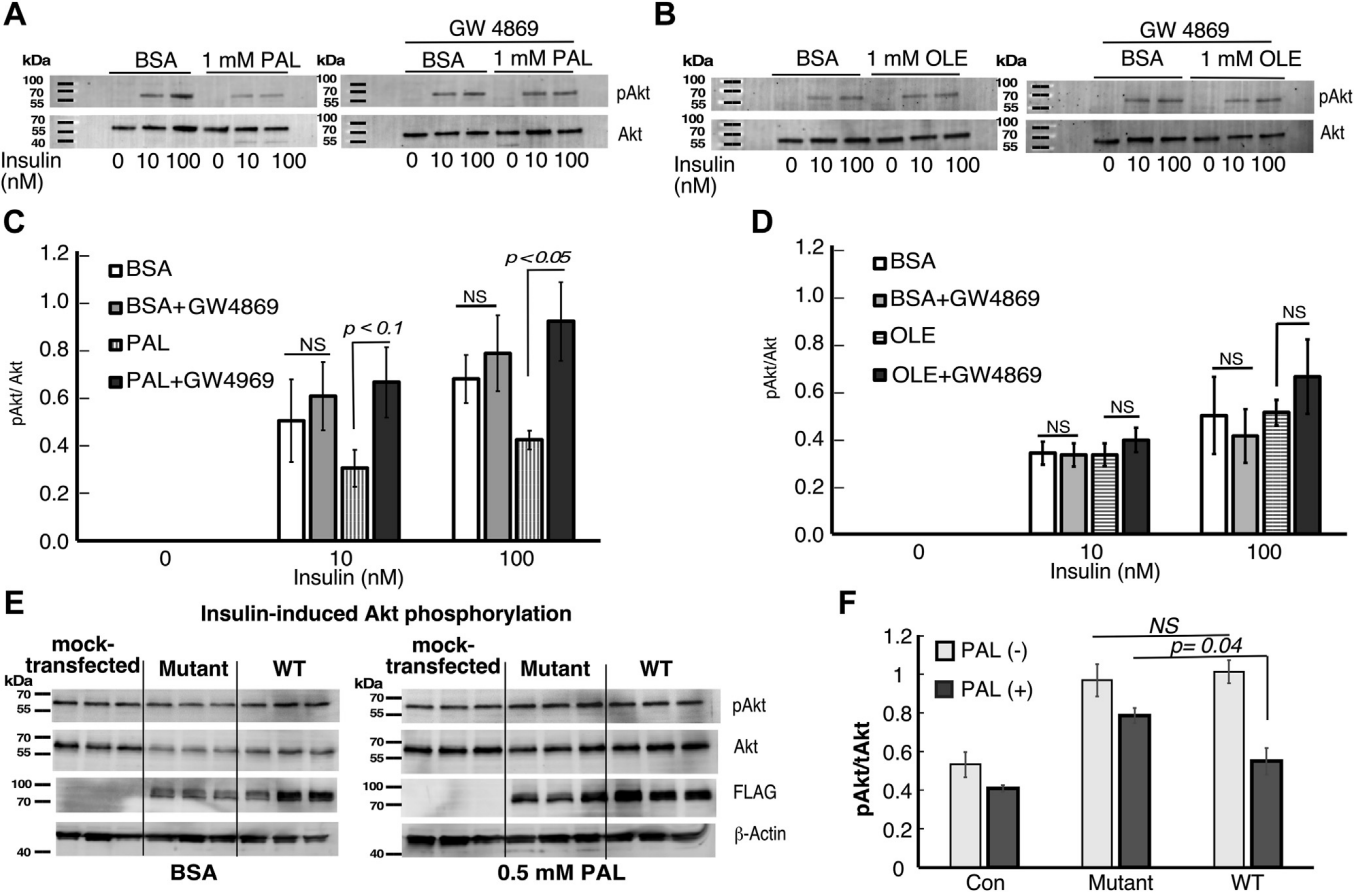
nSMase2 is required and sufficient for PAL-induced insulin resistance. A–D: insulin-induced Akt-1 phosphorylation in palmitate (A, C)- or oleate (B, D)-treated HepG2 cells in the presence or absence of nSMase2 inhibitor. HepG2 cells were treated with 1 mM fatty acids as indicated, delivered as a complex with BSA for 18 h. Cells were then stimulated with insulin at the indicated concentrations in the presence or absence of GW4869, an inhibitor of nSMase2 for 30 min. E, F: Overexpression of WT but not mutant nSMase2 potentiates PAL-induced suppression of insulin signaling*.* HepG2 cells were transfected with plasmids, expressing WT or mutant (^53,54,59,395,396^Cys-to-Ala) forms of nSMase2 for 48 h. Then the cells were treated with palmitic acid at suboptimal dose (0.5 mM) for 18 h followed by insulin (30 min, 100 nM). Akt-1 phosphorylation was analyzed by Western blotting and representative blots are shown (A, B, E). Quantification of the results was done by normalizing for the levels of total Akt (C, D, F). Data are average of three replicates and are representative of at least two independent experiments. nSMase2, neutral sphingomyelinase 2; PAL, palmitic acid.

An inhibitor of nSMase2, GW4869, was used to test whether the translocation of nSMase2 to the PM contributes to the onset of IR. GW4869 is a highly selective inhibitor of nSMase2 that has been used in vivo and in vitro to show the role for nSMase2 in biological processes (27, 53, 54). It also is the inhibitor of choice to suppress nSMase2-dependent exosome release. Indeed, the addition of GW4869 prior to and during insulin stimulation (for total of 45 min) completely prevented the suppression of Akt phosphorylation seen in PAL-pretreated cells (Fig. 6A, C). The inhibitor had no effect on the patterns of Akt activation seen in the OLE-treated cells (Fig. 6B, D). This outcome supports the proposed role of PAL-induced nSMase2 translocation in IR onset. The known mechanism of GW4869 action substantiates such conclusion further. GW4869 acts as a noncompetitive inhibitor in respect to the substrate SM but shows competitive characteristics toward the activator, phosphatidylserine (35). GW4869 seems to inhibit nSMase2 to a large extent by preventing the allosteric activation of the nSMase2 C-terminal catalytic domain following binding of the hydrophobic N-terminal domain to phosphatidylserine at the inner leaflet of the PM (29). Notably, this requirement of phosphatidylserine for activation distinguishes the nSMase2 from all other neutral SMases (55, 56) corroborating the specificity of the GW4869 effects seen herein. Nevertheless, an independent confirmation using a genetic approach to silence nSMase2 with siRNAi would further strengthen the conclusions.

In a parallel study, we attempted to silence nSMase2 via siRNAi. Unfortunately, we were not successful in effectively monitoring the efficacy of nSMase2 silencing in HepG2 cells due to the low level of nSMase2 expression, which prevented the rigorous employment of this alternative approach. Our results from an ongoing study with a newly developed mouse carrying liver-specific deletion of nSMase2 (currently prepared for a separate publication) provide evidence for a similar role of nSMase2 in obesity-induced hepatic IR.

This outcome indicates that an active nSMase2 is a required element in the mechanisms of PAL-induced IR. To test the opposite concept, that is, whether nSMase2 alone is sufficient to suppress insulin response, the ability of insulin to induce Akt phosphorylation was tested in nSMase2-overexpressing and mock-transfected HepG2 cells without PAL cotreatment. Despite having substantially higher nSMase2 activity, however, the nSMase2-overexpressing cells presented with similar insulin responsiveness as control cells (data not shown). Next, mock-transfected and NSMase-2–overexpressing HepG2 cells were treated with PAL at a suboptimal dose, that is, 0.5 mM. At this dose, PAL exerts only a small inhibitory effect (between 15% and 20%) on insulin-induced phosphorylation of Akt (Fig. 6E, F). This effect was substantially augmented however and reached statistical significance in HepG2 cells overexpressing the WT nSMase2. In contrast, HepG2 cells overexpressing the palmitoylation-defective mutant of nSMase2 exhibited a pattern of pAkt/Akt ratio like mock-transfected cells. The statistically nonsignificant trend of suppression seen in both, control and mutant transfected, PAL-treated cells as compared to nontreated likely represents the effects of the endogenous nSMase2. Apparently, the increased abundance of nSMase2 in the cells potentiated the suppressive effect of palmitate on Akt phosphorylation.

## Discussion

The studies reported here provide the first evidence that nSMase2-generated ceramide is a required element in the mechanism of IR in fat-laden hepatocytes. IR is characterized by the subdued activation of the proximal insulin signaling cascade, particularly Akt, which in turn leads to deregulation of downstream targets, like GSK3, PRAS40, Foxo1, and, consequently, the inability to downregulate gluconeogenesis during NAFLD. Saturated fats, notably palmitate, are the culprits for the onset of IR in hepatocytes. The known underlying mechanisms are either extrinsic and involve pro-inflammatory cytokines (IL-1β, TNFα, and IL-6) that are released by the Kupffer cells following lipotoxic death of hepatocytes or intrinsic and involve the cellular response to mitochondrial dysfunction and ER stress. IRAK-1, JNK, and NF-κB are thought to mediate both pathways (57, 58). Our study shows that PM-generated ceramide also affects Akt phosphorylation and ostensibly, might be the primary mechanism of IR onset.

During steatosis induced by an excess of PAL but not of the unsaturated OLE, nSMase2 undergoes a translocation to the PM, which is regulated via palmitoylation at 5 cysteines. Palmitoylation of the free SH group in cysteines is found frequently in signaling proteins, such as G-protein coupled receptors, ion channels, receptor tyrosine kinases, and others and serves to regulate protein transport and stability. In a few cases, the cellular levels of PAL have been shown to correlate with palmitoylation rates, for example, in the case of thioredoxin, CD36, Glut-4, and AMPA glutamate receptor (48, 49, 50). One of the key results of the present study is that nSMase2 is among these few proteins, which palmitoylation rates and consequently, PM localization, depends upon the cellular abundance of PAL.

At basal conditions, some nSMase2 molecules are already present at the PM, but the extent varies among the different hepatic cell types. In HepG2 and AML-12 cells, PM-associated nSMase2 is a minor fraction, while the majority is diffusely present in the cytoplasm and the Golgi. In primary mouse hepatocytes, a much greater fraction of the cellular nSMase2 is associated with the PM, even in the absence of excess PAL. Earlier, we found the same to be true for rat hepatocytes (15). It is noteworthy however that primary hepatocytes do not proliferate and are cultured on thick collagen (mouse) or in Matrigel (rat) matrix to maintain their differentiated status. Since the onset of growth arrest drives nSMase2 to the PM (14), the differences in the basal nSMase2 subcellular localization seen between HepG2/AML-12 cells (rapidly proliferating) and primary hepatocytes (highly differentiated, growth arrested) may reflect differences in the proliferation/differentiation status of these cells. Nonetheless, in all cell types investigated in this study, the elevated abundance of PAL was associated with further nSMase2 PM translocation, implying that stimulation of nSMase2 palmitoylation and PM translocation might be a uniform response of the liver to excess PAL.

SM is localized predominantly at the outer leaflet of the PM and does not undergo a spontaneous flip-flop across the membrane (45). Only recently, a peripheral myelin protein 2 was found to facilitate the trans bilayer movement of SM (46). Our studies join other recent investigations (59) in providing evidence that sufficient SM is in fact available as a substrate for the cytosolic leaflet-facing nSMase2. Moreover, the amounts of ceramide generated were apparently sufficient to exert a physiological effect and to suppress the insulin cascade. At least in HepG2 cells, the only cell lines examined in the colocalization studies, a generation of ceramide at the PM, was observed only in the PAL-treated cells; therefore, it is possible that the transport/generation of SM to serve as a substrate of nSMase2 at the inner leaflet is coordinately regulated with nSMase2 palmitoylation and translocation. Such scenario is likely because (i) excess palmitate also drives overall sphingolipid synthesis, including that of SM. Our experiments with ^3^H-palmitate confirmed that the flux though the SM synthetic pathway is stimulated by high PAL abundance. In such case, the capacity of the cells to accumulate ceramide at the PM following nSMase2 translocation might depend upon the simultaneous activation of the de novo pathway; (ii) protein palmitoylation typically happens in the Golgi and often serves to anchor palmitoylated proteins to preformed sphingolipid/cholesterol-rich platforms that are translocated to the PM via vesicular transport (60). Therefore, the assembly of lipid rafts, where the insulin receptors seemingly resides and which requires SM and glucosylceramide synthesis, might be coordinated with and/or affected by nSMase2 palmitoylation. The validity of such interactions remains to be investigated. At any rate, it is plausible that PM translocation of nSMase2 is a part of an integrated, sphingolipid-mediated cellular response to excess palmitate. This response includes on one hand, effects at the level of the PM caused by the local generation of ceramide via nSMase2 (i.e., lipid raft remodeling, direct suppression of akt phosphorylation) and on the other hand, effects at intracellular membranes brought about by the stimulation of de novo synthesis of ceramide (such as mitochondrial dysfunction, ER and oxidative stress, and lipotoxicity).

Various studies in the past have shown the capacity of ceramide to inhibit Akt. These earlier studies, however, have been based on the use of cell-permeable ceramide analogs (like C2-ceramide), bacterial SMase (that degrades SM in the outer leaflet of the PM), or inhibitors of de novo synthesis of ceramide, which exert broad effects on multiple cellular compartments. In this respect, our study is the first one to associate locally, nSMase2-generated ceramide to IR. Two proteins, the activity of which is influenced by direct binding to ceramide, have the capacity to suppress Akt phosphorylation: PKCζ, which also binds to Akt and negatively regulates its activation (61) and PP2A, which directly dephosphorylates Akt-1 at position S473 (62). None of these has been directly linked to nSMase2 so far. Our earlier studies in hepatocytes have shown that okadaic acid and siRNAi against PP2A catalytic subunit interfere with the mediatory role that nSMase2 plays in the IL-1β signaling cascade (36). Whether similar mechanisms are involved here, albeit likely, remains to be determined.

Noteworthy is also the observation that OLE fails to induce nSMase2 PM translocation despite inducing similar steatotic phenotype. This suggests that the nSMase2 translocation and the consequences it has on insulin signaling are not dependent upon steatosis per se. nSMase2 PM translocation is likely to be a characteristic of steatosis associated with PAL, which is the most abundant among all saturated fats found in the diet. This fact underlies the physiological significance of the observations reported herein because dietary saturated fatty acids have been associated with IR and the progression of simple steatosis to more aggressive stages of NAFLD. Therefore, the PM translocation of nSMase2 could serve to distinguish a benign from detrimental form of fatty liver. In conclusion, our study defines nSMase2 as novel component of the mechanism of IR in hepatocytes, which is cell autonomous, independent of inflammatory stimuli and lipotoxic death, and specifically driven by saturated fats.

## Data Availability

All data are contained within the manuscript.

## Supplemental data

This article contains supplemental data.

## Conflict of interest

The authors declare that they have no conflicts of interest with the contents of this article.

## Figures and Tables

**Supplemental Fig. 5:**